# The association between body mass index and vulvar and vaginal cancer incidence: findings from a large Norwegian cohort study

**DOI:** 10.1007/s10552-024-01930-z

**Published:** 2024-10-27

**Authors:** Dagfinn Aune, Marie Nordsletten, Tor Åge Myklebust, Trude Eid Robsahm, Bjørn Steen Skålhegg, Tom Mala, Sheraz Yaqub, Usman Saeed

**Affiliations:** 1 Department of Research, Cancer Registry of Norway, Norwegian Institute of Public Health, Oslo, Norway; 2 Department of Nutrition, Oslo New University College, Oslo, Norway; 3 Department of Epidemiology and Biostatistics, School of Public Health, White City Campus, 90 Wood Lane, London, W12 0BZ UK; 4 Department of Gastrointestinal and Paediatric Surgery, Oslo University Hospital, Oslo, Norway; 5 Department of Registration, Cancer Registry of Norway, Norwegian Institute of Public Health, Oslo, Norway; 6 Department of Research and Innovation, Møre and Romsdal Hospital Trust, Ålesund, Norway; 7 Division for Molecular Nutrition, Institute of Basic Medical Sciences, University of Oslo, Oslo, Norway; 8 Institute for Clinical Medicine, University of Oslo, Oslo, Norway

**Keywords:** Body mass index, Vulvar cancer, Vaginal cancer, Cohort

## Abstract

**Background:**

There is limited evidence of potential associations between body mass index (BMI) and risk of vulvar and vaginal cancer. We explored these associations in a large cohort of Norwegian women.

**Methods:**

The analytical dataset included 889,441 women aged 16–75 years at baseline in 1963–1975. Multivariable Cox regression analyses were used to estimate hazard ratios (HRs) and 95% confidence intervals (CIs) for the associations between BMI and vulvar and vaginal cancer incidence.

**Results:**

During 30.1 million person-years of follow-up, 1748 incident vulvar and 408 incident vaginal cancer cases occurred. The HRs (95% CIs) for vulvar cancer for a BMI of 15- < 18.5, 18.5- < 25, 25- < 30, 30- < 35, ≥ 35 were 0.62 (0.38–1.01), 1.00 (reference), 1.23 (1.10–1.40), 1.43 (1.23–1.66) and 1.72 (1.35–2.20, p_trend_ < 0.001), and per 5 kg/m^2^ increment was 1.20 (1.13–1.26). The corresponding HRs (95% CIs) for vaginal cancer were 1.05 (0.52–2.15), 1.00, 0.89 (0.71–1.12), 0.95 (0.68–1.34), and 2.01 (1.29–3.13, p_trend_ < 0.001), respectively, and per 5 kg/m^2^ was 1.11 (0.99–1.25). The HR (95% CI) per 5 kg/m^2^ increase in BMI at ages 16–29 was 1.28 (1.07–1.54, *n* = 250 cases) for vulvar and 1.53 (1.11–2.11, *n* = 66 cases) for vaginal cancers. The HR (95% CI) per 5 kg/m^2^ for early-onset (< 50 years age at diagnosis) vulvar cancer was 0.92 (0.66–1.28, *n* = 87 cases) and 1.70 (1.05–2.76, *n* = 21 cases) for vaginal cancer.

**Conclusion:**

These results further support the associations between higher BMI and increased risk of vulvar and vaginal cancers, with suggestive stronger associations between BMI in early adulthood for both cancers and for early-onset vaginal cancer. Further studies are needed to elucidate these findings and investigate the underlying mechanisms.

## Introduction

Vulvar and vaginal cancers are rare with an overall incidence rate globally of 0.9 cases per 100,000 person-years and 0.4 cases per 100,000 person-years, respectively, and a total of 45,000 and 18,000 cases occurred globally in 2020 [1]. Risk factors include human papilloma virus (HPV) infection [2, 3], sexual habits [2–4], various pre-cancerous lesions [3, 5], genital warts [2–4, 6–8], vulvar lichen sclerosis [3], conditions associated with immunosuppression such as HIV [3], systemic lupus erythematosus [9] and organ transplantation [3, 10, 11], diethylstilbestrol use [12], alcoholism [13] and smoking [2, 4, 14].

Excess weight is a risk factor for 12 cancers according to the World Cancer Research Fund [15], however, limited data is available on adiposity and risk of vulvar and vaginal cancers. The Me–Can study found a 2.3-fold increase in risk of vulvar cancer and a non-significant 2.1-fold increased risk of vaginal cancer when comparing women with obesity with those with normal weight [16]. An analysis in the NIH-AARP Diet and Health Study found a 62% increase risk of vulvar cancer when comparing women with obesity vs. normal weight [14], and the Million Women's Study found similarly a 71% increase in risk among women with obesity vs. normal weight [17]. Recently, a large pooled analysis of 2 million Swedish women reported a HR of 2.43 (1.88–3.14) for vulvar cancer for women with obesity vs. normal weight and 1.42 (1.29–1.55) per 5 kg/m^2^ increase in BMI and a HR of 1.22 (0.97–1.55) for vaginal cancer per 5 kg/m^2^ increase in BMI, providing further support for these observations [18]. A registry-based cohort study from Denmark reported a 1.67-fold increased risk of both vulvar and vaginal cancers among women with a hospital diagnosis of obesity [19]. In addition, two case–control studies have reported 2.5-fold [20] to 2.9-fold [21] increases in vulvar cancer risk with high BMI. We are not aware of other published studies on adiposity and risk of these cancers. Because of the low incidence, very large cohort studies are needed to study these cancer types. We investigated the associations between measured BMI and vulvar and vaginal cancer risk in a large cohort of Norwegian women who participated in the Norwegian Tuberculosis Screening Program.

## Methods

The Norwegian Tuberculosis Screening Program (NTSP) was a nationwide screening program for tuberculosis between 1943 and 1999 in Norway. A time-restricted extended nationwide and unselected mass survey was conducted in 1963–1975 within the NTSP, recruiting about half the total Norwegian population of four million individuals. Those participating in this extended mass survey were eligible for inclusion in the present study.

Weight and height measurements were obtained from the NTSP mass survey during 1963–1975 [22]. Weight and height were measured and registered electronically by health professionals at baseline. BMI was estimated by dividing weight in kg with height^2^ in meters (BMI = weight/height^2^). The current study focused on adult BMI. The NTSP data were linked to data on cancer diagnoses from the Cancer Registry of Norway (CRN) using the personal identification number assigned to all Norwegian citizens. The data from the CRN is documented high quality registry with high quality data and close to complete national data [23]. Information on vital status and date of death and emigration was obtained by linkage to the National Population Register that is continuously updated.

## Study population

The NTSP survey included a total of 1,911,598 Norwegians (aged 7–99 years). The current analysis excluded all men (*n* = 918,000), those aged < 16 years and > 75 years (*n* = 68,844), those with missing data on weight or height (*n* = 1821), those with BMI < 15 or > 50 kg/m^2^ (*n* = 393), short stature (< 150 cm) (*n* = 17,760), those diagnosed with cancer (except cutaneous basal cell carcinoma) at baseline or within the first year of follow-up or with uncertain cancer diagnosis (*n* = 15,021), and those with no follow-up time (*n* = 318). After all exclusions were made, the current analysis included 889,441 women aged 16–75 years at baseline.

## Outcome and follow-up data

Cancer diagnoses were obtained by linkage to the CRN using the International Classification of Diseases version 10 (ICD-10) codes. Vulvar and vaginal cancer cases were identified by ICD-10 codes C51 and C52, respectively. Individuals were followed from the NTSP screening date until date of vulvar or vaginal cancer diagnosis, 75 years of age, death, emigration, or the end of follow-up (December 31, 2018), whichever came first.

## Statistical methods

Multivariable Cox proportional hazards regression models were used to estimate HRs (95% CIs) for the association between BMI and vulvar and vaginal cancers. The proportional hazards assumption was tested using Schoenfeld residuals test and no deviation was observed for vulvar (*p* = 0.09) or vaginal cancer (*p* = 0.57). The analyses were adjusted for age groups at the time of screening, with age as the underlying time scale. BMI was categorized by standard cut-off points 15– < 18.5, 18.5– < 25, 25– < 30, ≥ 30. Obesity was further categorized into 30– < 35, and ≥ 35, to assess the impact of more extreme levels of obesity on vulvar and vaginal cancer risk. Linear trends were explored by replacing the BMI category with the median BMI value within each defined category and entering this variable as a continuous variable in the models. We also analysed the association per 5 kg/m^2^ increase in BMI. To explore potential nonlinear associations, BMI was also modelled using restricted cubic splines with five degrees of freedom. We tested for nonlinearity by including a quadratic term of BMI (BMI^2^) in the models.

Sensitivity analyses were made by excluding the first 5 years of follow-up to further take into account reverse causation biases. Analyses were conducted among individuals aged 16–29 years at the time of screening to assess any potential association between BMI in early adulthood and these cancers. Furthermore, we explored the association between BMI and early-onset (age < 50 years at diagnosis) vulvar and vaginal cancer.

## Results

The analytical cohort included 889,441 women aged 16–75 years at baseline (Table 1). The mean follow-up was 32.5 (SD 14.8) years and over 30.1 million person-years accrued a total of 1748 vulvar and 408 vaginal cancer cases. The mean age at diagnosis was 72.3 years for vulvar cancer and 70.9 years for vaginal cancer.

**Table 1:** Characteristics of the participants in the Norwegian tuberculosis screening program

	Women
Study cohort	889,441
Age at baseline (years)	43.1 (16.5)
Age group 16–29 years	233,098 (26.3%)
Height (cm)	162.5 (5.8)
Weight (kg)	65.6 (11.4)
BMI (kg/m^2^)	24.9 (4.4)
BMI categories	
Underweight (15– < 18.5)	23,693 (2.7%)
Normal weight (18.5– < 25.0)	491,290 (55.2%)
Overweight (25– < 30.0)	261,499 (29.4%)
Obese, all (≥ 30.0)	112,959 (12.7%)
Obese grade 1 (30– < 35.0)	87,491 (9.8%)
Obese grade 2 (≥ 35.0)	25,468 (2.9%)

Values are means (SDs) for continuous variables and numbers (percentages for categorical variables)

**Fig. 1:**
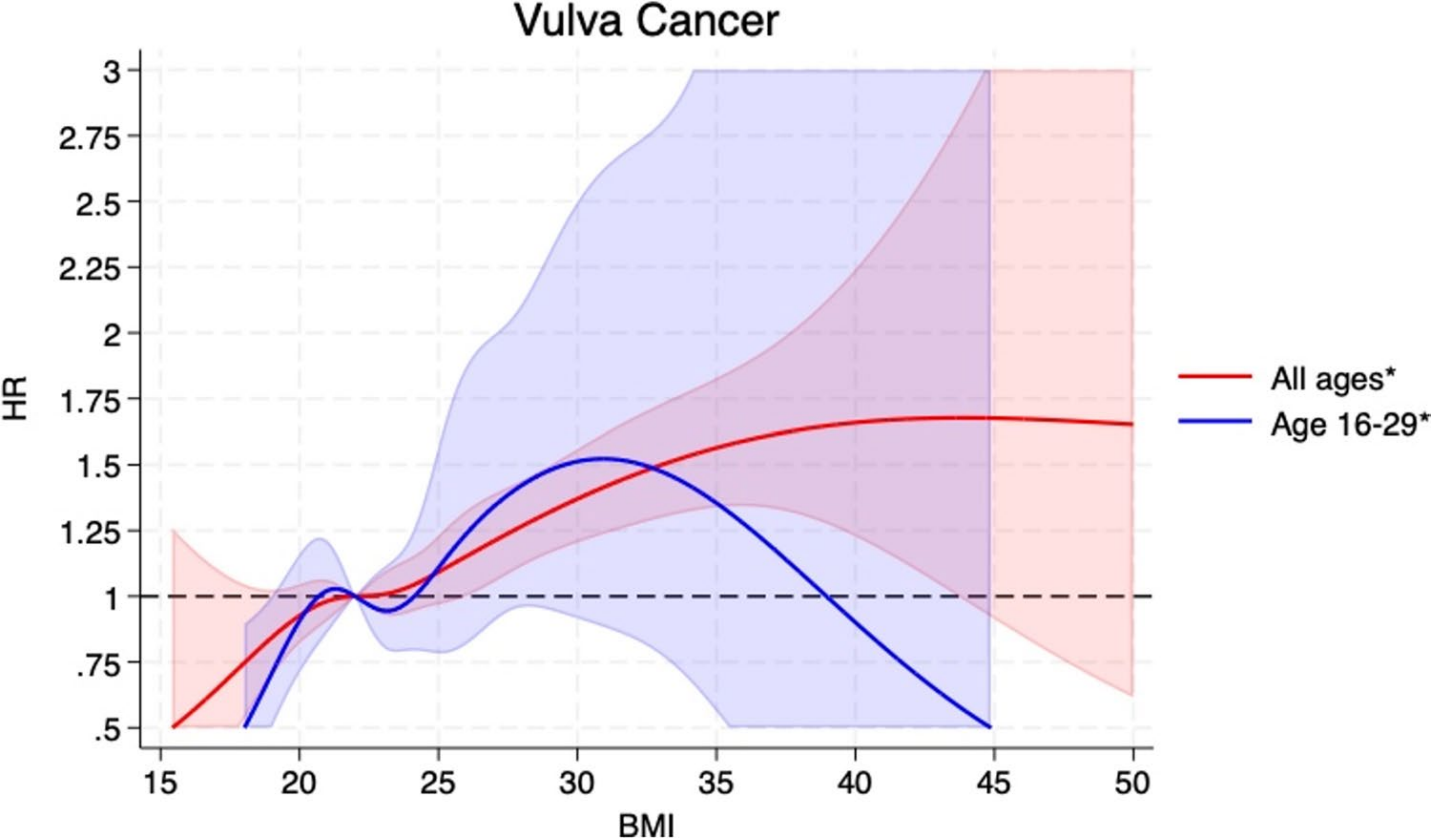
BMI and vulvar cancer, restricted cubic splines

**Fig. 2:**
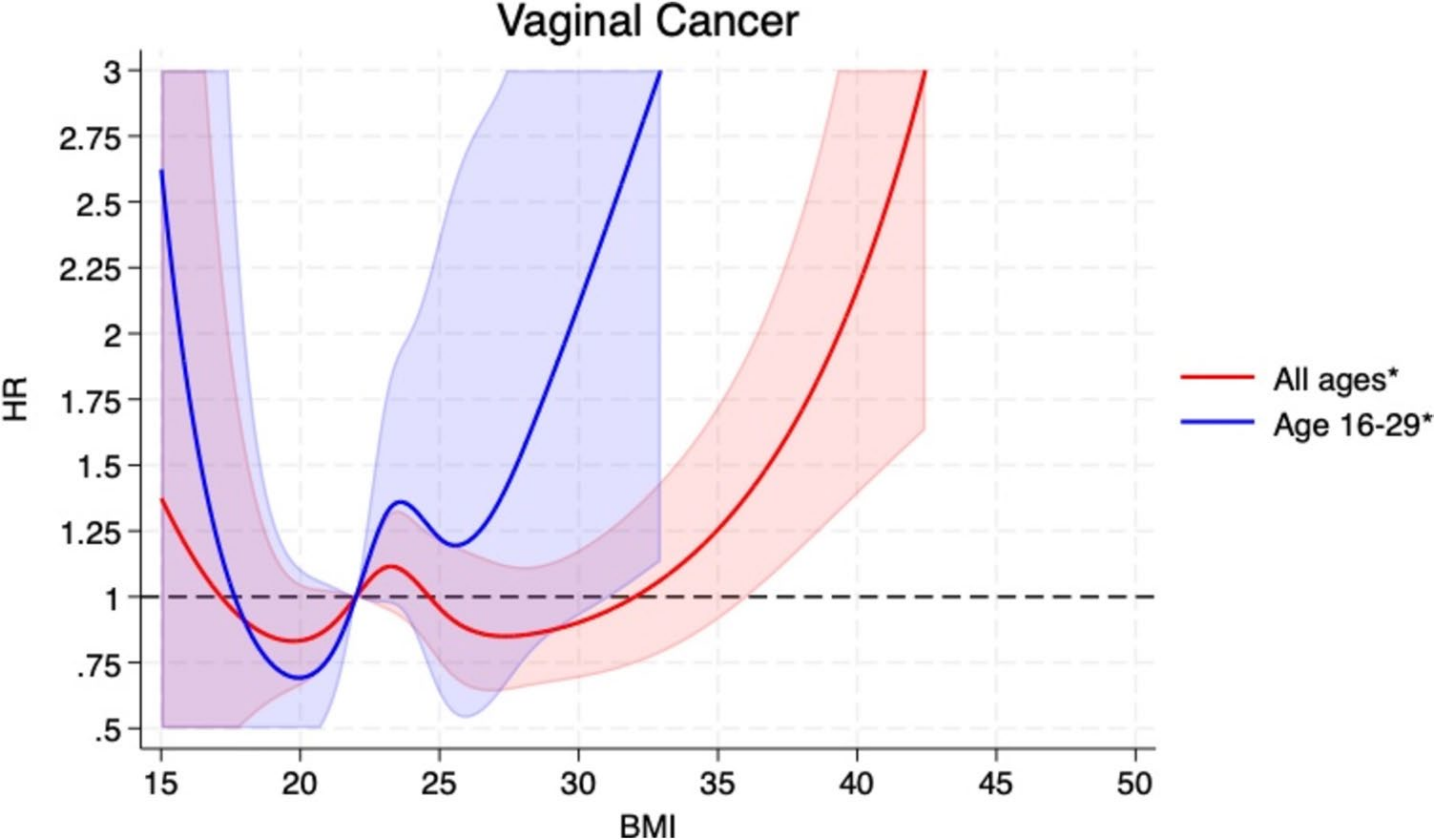
BMI and vaginal cancer, restricted cubic splines

Compared to women with BMI 18.5– < 25, the HRs (95% CIs) of vulvar cancer among those with a BMI of 15– < 18.5, 25– < 30, and ≥ 30 were 0.62 (0.38–1.01), 1.23 (1.10–1.40), and 1.49 (1.30–1.70), respectively (Table 2). When categorized according to obesity grade 1 (BMI 30– < 35) and grade 2 (≥ 35), the respective HRs were 1.43 (1.23–1.66) and 1.72 (1.35–2.20, p_trend_ < 0.001), and when analysed per 5 kg/m^2^ increment the HR was 1.20 (1.13–1.26) (Table 2). The corresponding HRs (95% CIs) for vaginal cancer were 1.05 (0.52–2.15), 0.89 (0.71–1.12), 1.17 (0.88–1.57), 0.95 (0.68–1.34), and 2.01 (1.29–3.13, p_trend_ < 0.001), respectively, and the HR per 5 kg/m^2^ was 1.11 (0.99–1.25) (Table 3). These positive associations persisted when excluding the first 5 years of follow-up, however, the association was weaker for vulvar cancer while the association with vaginal cancer showed similar strength (Table 2 and 3). The positive associations were also observed in analyses using restricted cubic splines and there was no evidence of nonlinearity for vulvar cancer (p_nonlinearity_ = 0.66) (Fig. 1), but some evidence of nonlinearity for vaginal cancer (p_nonlinearity_ = 0.02) (Fig. 2).

**Table 2:** Hazard ratios (HRs) and 95% confidence intervals (95% CIs) for the association between body mass index categories and the risk of vulvar cancer

	Body mass index							
	15– < 18.5	18.5– < 25.0	25.0– < 30.0	30.0– < 35.0	≥ 35.0	≥ 30.0	Per 5 kg/m^b^	p_trend_
Person-years	961,436	18,693,695	7,659,480	2,184,800	595,488	2,780,288	30,094,901	
Participants	23,693	491,290	261,499	87,491	25,468	112,959	889,441	
Cases	17	816	619	226	70	296	1748	
HR (95% CI)^a^	0.62 (0.38–1.01)	1.00	1.23 (1.10–1.40)	1.43 (1.23–1.66)	1.72 (1.35–2.20)	1.49 (1.30–1.70)	1.20 (1.13–1.26)	< 0.001
Person-years	959,651	18,635,803	7,616,481	2,170,144	591,045	2,761,189	29,973,126	
Participants	23,557	487,040	257,477	85,879	24,942	110,821	878,895	
Cases	16	794	583	201	57	258	1651	
HR (95% CI)^2^	0.60 (0.36–0.98)	1.00	1.19 (1.07–1.33)	1.31 (1.12–1.53)	1.46 (1.11–1.91)	1.34 (1.16–1.54)	1.15 (1.08–1.22)	< 0.001

^a^Adjusted for age

^b^Adjusted for age, excluding first 5 years of follow-up

**Table 3:** Hazard ratios (HRs) and 95% confidence intervals (95% CIs) for the association between body mass index categories and the risk of vaginal cancer

	Body mass index							
	15– < 18.5	18.5– < 25.0	25.0– < 30.0	30.0– < 35.0	≥ 35.0	≥ 30.0	Per 5 kg/m^2^	p_trend_
Person-years	961,488	18,698,150	7,662,138	2,185,591	595,696	2,781,288	30,103,065	
Participants	23,693	491,290	261,499	87,491	25,468	112,959	889,441	
Cases	8	220	118	40	22	62	408	
HR (95% CI)^a^	1.05 (0.52–2.15)	1.00	0.89 (0.71–1.12)	0.95 (0.68–1.34)	2.01 (1.29–3.13)	1.17 (0.88–1.57)	1.11 (0.99–1.25)	< 0.001
Person-years	959,689	18,639,886	7,618,642	2,170,783	591,195	2,761,979	29,980,198	
Participants	23,557	487,040	257,477	85,879	24,942	110,821	878,895	
Cases	7	213	110	38	21	59	389	
HR (95% CI)^b^	0.96 (0.45–2.05)	1.00	0.85 (0.67–1.08)	0.93 (0.66–1.33)	1.98 (1.26–3.12)	1.15 (0.86–1.55)	1.10 (0.98–1.24)	< 0.001

^a^Adjusted for age

^b^Adjusted for age, excluding first 5 years of follow-up

The HRs (95% CIs) for the association between a 5 kg/m^2^ increment in BMI at ages 16–29 years and vulvar cancer (*n* = 250 cases) was 1.28 (1.07–1.54) and for vaginal cancer (66 cases) was 1.53 (1.11–2.11) (Table 4). The HRs (95% CIs) per 5 kg/m^2^ increment in BMI for early-onset vulvar cancer (*n* = 87 cases) was 0.92 (0.66–1.28), and for early-onset vaginal cancer 1.70 (1.05–2.76, *n* = 21 cases) (Table 5).

**Table 4:** BMI at age 16–29 years and vulvar and vaginal cancer

	Vulvar cancer							
	Body mass index							
	15– < 18.5	18.5– < 25.0	25.0– < 30.0	30.0– < 35.0	≥ 35.0	≥ 30.0	Per 5 kg/m^2^	p_trend_
Person-years	717,492	8,655,321	1,292,114	187,871	41,989	229,860	10,894,789	
Participants	15,514	184,885	27,659	4100	940	5040	233,098	
Cases^2^	–	–	–	–	–	–	250	
HR (95% CI)	0.34 (0.14–0.83)	1.00	1.42 (1.02–1.96)	1.29 (0.57–2.92)	0.96 (0.13–6.84)	1.23 (0.60–2.62)	1.28 (1.07–1.54)	0.008

	Vaginal cancer							
	Body mass index							
	15– < 18.5	18.5– < 25.0	25.0– < 30.0	30.0– < 35.0	≥ 35.0	≥ 30.0	Per 5 kg/m^2^	p_trend_
Person-years	717,482	8,656,686	1,292,452	187,894	41,985	229,880	10,896,501	
Participants	15,514	184,885	27,659	4100	940	5040	233,098	
Cases^a^	–	–	–	–	–	–	66	
HR (95% CI)	1.06 (0.38–2.94)	1.00	1.16 (0.57–2.37)	1.74 (0.42–7.17)	7.78 (1.89–32.01)	2.85 (1.02–7.89)	1.53 (1.11–2.11)	0.047

^a^Categorical case numbers have been suppressed because of low numbers (< 5) in some categories

**Table 5:** BMI and risk of early-onset vulvar and vaginal cancer

	Early-onset vulvar cancer					p_trend_
	Body mass index					
	15– < 18.5	18.5– < 25.0	25.0– < 30.0	≥ 30.0	Per 5 kg/m^2^	
Person-years	503,360	7,162,251	1,517,827	359,250	9,542,690	
Participants	20,405	375,309	118,378	34,731	548,823	
Cases^a^	–	–	–	–	87	
HR (95% CI)	0.22 (0.03–1.58)	1.00	1.05 (0.61–1.82)	-	0.92 (0.66–1.28)	< 0.001

	Early-onset vaginal cancer					p_trend_
	Body mass index					
	15– < 18.5	18.5– < 25.0	25.0– < 30.0	≥ 30.0	Per 5 kg/m^2^	
Person-years	503,359	7,162,497	1,517,881	359,235	9,542,973	
Participants	20,405	375,309	118,378	34,731	548,823	
Cases^a^	–	–	–	–	21	
HR (95% CI)	1.16 (0.15–9.00)	1.00	2.82 (1.08–7.34)	1.73 (0.22–13.73)	1.70 (1.05–2.76)	< 0.001

^a^Categorical case numbers have been suppressed because of low numbers (< 5) in some categories

## Discussion

We found positive associations between higher BMI and risk of vulvar and vaginal cancers, with 23%, 43% and 72% increases in risk of vulvar cancer with overweight, grade 1 and grade 2 obesity, respectively, and a 101% increase in risk of vaginal cancer with grade 2 obesity vs. normal weight. These associations persisted in sensitivity analyses excluding the first 5 years of follow-up for both cancers. The associations with BMI in early adulthood (ages 16–29 years) were more pronounced with a 28% increase in risk for vulvar cancer and a 53% increase in risk for vaginal cancer per 5 kg/m^2^ increment in BMI, respectively. The association with early-onset cancer (< 50 years age) was null for vulvar cancer, but further strengthened for vaginal cancer. These latter analyses, however, were based on a low number of cases.

Our findings are consistent with the results of the Me-Can study, which reported a 2.4-fold and a non-significant 2.1-fold increased risk of vulvar and vaginal cancer, respectively, when comparing women with obesity with those with normal weight [16]. A registry-based cohort study from Denmark reported a 1.67-fold increased risk for both vulvar and vaginal cancers with a hospital diagnosis of obesity [19]. Similarly, in the NIH-AARP Diet and Health Study, a 62% increased risk of vulvar cancer was observed among women with obesity vs. normal weight [14], and the Million Women's Study reported a 71% increase in risk for the same comparison [17]. Recently, a large pooled analysis of 2 million Swedish women reported a HR of 2.43 (1.88–3.14) for vulvar cancer for women with obesity vs. normal weight and a HR of 1.42 (1.29–1.55) per 5 kg/m^2^ increase in BMI and for vaginal cancer a HR of 1.22 (0.97–1.55) per 5 kg/m^2^ increase in BMI, providing further support for these observations [18]. Lastly, two case–control studies reported strong positive associations between BMI and vulvar cancer [20, 21]. We are not aware of previous studies on BMI in young adulthood and risk of these cancer sites or on BMI and early-onset vulvar and vaginal cancers. Overweight and obesity tends to track quite strongly from early life into adulthood [24–27], thus it is possible that longer-term exposure to excess weight may be most important or that a certain time window of exposure may be particularly relevant for the development of these cancers. Further studies are needed to address this question as the number of cancer cases in these subsets of the cohort was relatively low.

The biological mechanism(s) that could explain these associations remains unclear and somewhat speculative. Cross-sectional and case–control studies have reported a positive association between overweight or obesity and prevalence of genital lichen sclerosus [28, 29], which again is strongly associated with increased risk of vulvar cancer [30–34]. A few studies have reported positive associations between type 2 diabetes or blood glucose levels and risk of vulvar [16, 35], and vaginal [16, 35] cancers, however, not all studies showed clear associations [14, 17, 36–38]. Adiposity is strongly associated with increased risk of type 2 diabetes [39], thus it is possible insulin resistance may be involved in the development of these cancers. Women with diabetes have been observed to be at increased risk of genital warts [40] and vaginitis [41], conditions that are associated with increased risk of vulvar and/or vaginal cancer [4, 6, 7, 42], however, women with overweight or obesity have been reported to have lower prevalence of genital warts [43] and high-risk HPV infection [43, 44] and similar risk of incident high-risk HPV infection [45] compared to normal weight women, casting some doubt on this possibility. Adiposity could also impair the ability to self-examine and identify early stages of vulvar cancer. Although hormonal factors are known to be important for other gynaecological cancers, use of oral contraceptives, hormone use and other hormone-related factors have not been strongly or consistently associated with vulvar cancer risk [3]. Further studies are needed to clarify the underlying mechanism(s) for the observed associations between adiposity and vulvar and vaginal cancer risk.

Strengths of the study include the large sample size, which provided sufficient statistical power to investigate the association between BMI and these rare cancer types. Weight and height were measured by healthcare professionals eliminating potential errors due to self-report. Linkages to well recognized national cancer and death registries with close to complete data limits errors in outcome assessment. Furthermore, up to 50 years follow-up with minimal attrition minimize potential bias due to loss to attrition. The study cohort covered approximately half the Norwegian population at the time it was conducted making findings most likely generalisable to the population at large at the time of the study. The main limitation of our study is no information on other relevant risk factors for development of vulvar and vaginal cancer including smoking, HPV and HIV infection. Smoking has been reported an important risk factor for development of vulvar and vaginal cancer [14, 46]. On the other hand, smokers tend to have lower BMI than non-smokers [47, 48], and potential confounding from smoking would most likely cause an underestimation of the observed associations between BMI and vulvar and vaginal cancer risk. Similarly, high-risk HPV infection is less prevalent among women with higher BMI [43, 44], so any confounding by HPV infection would also most likely lead to underestimation of the associations. Other cohort studies with more detailed adjustments for confounding factors report positive associations [14, 16, 17] in line with our observations, suggesting that the findings of this study may be less likely fully explained by confounding. During the long follow-up the weight of the participants may have changed. We did not have repeated assessments of anthropometric measures and were therefore not able to take any changes in weight trajectories into account. Given the general increase in adiposity in the Norwegian population over time [49] it is possible that part of the observed associations could be driven by weight gain.

In conclusion, we found that adiposity was associated with increased vulvar and vaginal cancer risk. Additional large cohort studies with more detailed adjustments for confounders are needed to clarify these associations and the underlying mechanisms.

## Data Availability

These data are not available for sharing publicly, but require applications for data access and ethical approval.
